# Genome-Wide DNA Methylation Profiling Reveals Low Methylation Variability in Moyamoya Disease

**DOI:** 10.1007/s12975-024-01299-w

**Published:** 2024-10-02

**Authors:** Kikutaro Tokairin, Masaki Ito, Alex G. Lee, Mario Teo, Shihao He, Michelle Y. Cheng, Gary K. Steinberg

**Affiliations:** 1 Department of Neurosurgery, Stanford University School of Medicine, 1201 Welch Road, Stanford, CA 94305 USA; 2 Stanford Stroke Center, Stanford University School of Medicine, Stanford, CA USA; 3 Division of Hematology and Oncology, Department of Pediatrics, University of California, San Francisco, CA USA; 4 Department of Neurosurgery, Peking Union Medical College Hospital, Peking, China

**Keywords:** Moyamoya disease, DNA methylation, Methylation variability, Cerebrovascular disorder, Stroke

## Abstract

Moyamoya disease (MMD) is a chronic cerebrovascular disorder that can lead to stroke and neurological dysfunctions. Given the largely sporadic nature and the role of gene-environment interactions in various diseases, we examined epigenetic modifications in MMD. We performed genome-wide DNA methylation using Illumina 850 K Methylation EPIC BeadChip, in two racially distinct adult female cohorts: a non-Asian cohort (13 MMD patients and 7 healthy controls) and an Asian cohort (14 MMD patients and 3 healthy controls). An additional external cohort with both sexes (females: 5 MMD patients and 5 healthy controls, males: 5 MMD patients and 5 healthy controls) was included for validation. Our findings revealed strikingly low DNA methylation variability between MMD patients and healthy controls, in both MMD female cohorts. In the non-Asian cohort, only 6 probes showed increased variability versus 647 probes that showed decreased variability. Similarly, in the Asian cohort, the MMD group also displayed a reduced methylation variability across all 2845 probes. Subsequent analysis showed that these differentially variable probes are located on genes involved in key biological processes such as methylation and transcription, DNA repair, cytoskeletal remodeling, natural killer cell signaling, cellular growth, and migration. These findings mark the first observation of low methylation variability in any disease, contrasting with the high variability observed in other disorders. This reduced methylation variability in MMD may hinder patients’ adaptability to environmental shifts, such as hemodynamic stress, thereby influencing vascular homeostasis and contributing to MMD pathology. These findings offer new insights into the mechanisms of MMD and potential treatment strategies.

**Supplementary Information:**

The online version contains supplementary material available at 10.1007/s12975-024-01299-w.

## Introduction

Moyamoya disease (MMD) is a rare, idiopathic cerebrovascular disorder affecting both children and adults. MMD is characterized by chronic and progressive narrowing of the cerebral arteries and the formation of abnormal network of collateral blood vessels [1, 2]. The narrowed arteries can lead to blockage and eventually cause stroke. To date, there is no known pharmacological treatment for MMD, and the only effective treatment is invasive revascularization surgery. Thus, understanding the mechanisms of MMD pathogenesis is an important step toward developing future non-surgical treatments to stop or slow the progression of this disease.

Approximately 10–15% of MMD cases are familial cases [3, 4]. The pathological mechanisms underlying MMD are still largely unknown. Genetic studies identified RNF213 (also known as mysterin) as a major susceptibility gene for MMD [5, 6], and this is particularly significant in East Asian populations. MMD is more common in East Asia, approximately 10 times higher than the incidence in Europe and North America (0.09 per 100,000 individuals) [7, 8]. In Caucasians, no equivalent susceptibility allele has been identified to date. Mutations in other genes such as DIAPH1, ACTA2, BRCC3, and GUCY1A3 have been associated mostly with moyamoya syndrome or related cerebral arteriopathies [9–12]. The low penetrance of MMD in patients with RNF213 mutations strongly suggests the involvement of other factors, which could be genetic/epigenetic, circulating, and/or environmental. Elevation of secreted factors in the plasma and the cerebrospinal fluid (CSF) of MMD patients has been reported, including various inflammatory cytokines, chemokines, growth factors, and angiogenetic mediators, such as vascular endothelial growth factor (VEGF), basic fibroblast growth factor (bFGF), platelet derived growth factor (PDGF), and plasminogen activator inhibitor 1 (PAI-1) [13–16]. A few studies have used transcriptome approaches to profile gene expression changes in MMD patient’s peripheral blood, smooth muscle progenitor cells (SPCs), and MMD iPSC-derived ECs and VSMCs. These studies reported dysregulation in genes involved in extracellular matrix (ECM) organization, immune/inflammatory responses, and cellular functions like migration, adhesion, and vascular development [17–20].

Considering that MMD is mostly sporadic and gene-environment interactions play important roles in many diseases, this has prompted us investigations into epigenetic changes in MMD. To date, there have been few reports on epigenetic regulation in MMD patients, such as chromatin remodeling and DNA methylation [21]. DNA methylation is a well-established epigenetic phenomenon that involves the transfer of a methyl group to the 5′-cytosine (C) base in a CpG dinucleotide, influencing gene transcription in response to environmental factors [22]. Such modifications can impact the stability of gene expression and have been linked to a range of diseases, including cancer, diabetes, immune disorders, and neurodegenerative and cardiovascular diseases [21–27]. DNA methylation research has been largely focused on examining differential methylation (DM), which compares mean DNA methylation levels between groups. However, increasing evidence suggests that DNA methylation variability (DV), comparing the methylation variability patterns between groups, may also affect disease susceptibility [28–32]. DM and DV provide different insights into how methylation patterns are altered, and both are important for understanding the role of epigenetics in health and disease.

In this study, we investigated genome-wide DNA methylation changes in MMD using Illumina 850 K Methylation EPIC array. We analyzed differential methylation (DM) and differential variability (DV) between MMD and healthy controls across two racially distinct cohorts. Our data revealed significant differences in methylation variability in MMD patients, and this was validated with an external cohort. We identified a panel of unique differentially variable probes (DVPs) that could be instrumental in the molecular mechanisms driving MMD pathogenesis and progression.

## Material and Methods

### Study Subjects

This study investigated two cohorts: (1) a non-Asian cohort consisting of 13 adult female MMD patients and 7 healthy adult female controls, primarily of Caucasian descent, including one healthy control of Hispanic ethnicity; (2) an Asian cohort including 14 Asian adult female MMD patients and 3 healthy adult female controls from diverse Asian backgrounds. Both groups were self-reported for race/ethnicity. The selection criteria are as follows: These patients were carefully selected from a large cohort of nearly 800 moyamoya patients treated at Stanford Moyamoya Center between 1991 and 2014 with detailed analyses of the patients’ demographics, treatment types, and outcomes previously published [33]. To increase the validity of the epigenetic analyses and to minimize the heterogeneity within moyamoya patients, strict selection criteria were applied which includes the following: (a) female; (b) younger patients (< 50 years old); (c) bilateral moyamoya disease; (d) non-familial moyamoya; (e) ischemic-type moyamoya disease (TIA symptoms or patients with radiological ischemia); (f) patients with moyamoya syndrome or quasi-moyamoya associated with known conditions such as sickle cell disease, Down syndrome, neurofibromatosis-1, or autoimmune diseases were excluded [12]; and (g) hemorrhagic presentation patients were excluded. MMD diagnosis was based on consensus criteria [34], with patients eligible for cerebral revascularization surgery due to ischemic symptoms or intractable headache with hemodynamic reserve impairment, and Suzuki’s angiographical stage was between 2 and 4 as shown in Table 1. To minimize confounding factors (e.g., age, cardiovascular risk factors) between moyamoya cases and controls, we included only younger female patients. Additionally, patients with diabetes and hyperlipidemia were excluded from the study. Consent from all participants at Stanford University were obtained, and whole blood samples were collected prior to surgery and anesthesia, which typically occurred within a year of symptom onset for the moyamoya patients included in this study. This study was approved by the Institutional Review Board of the Stanford University School of Medicine (IRB-12625). The external cohort for validation is as follows: Raw data from a previously published paper [21] was kindly provided by Dr. Shihao He, Dr. Yuanli Zhao, and Dr. Rong Wang at the Department of Neurosurgery, Beijing Tiantan Hospital, Beijing, China. This cohort consists of 10 MMD patients (*n* = 5 males, *n* = 5 females) and 10 healthy controls (*n* = 5 males, *n* = 5 females), all of Chinese Han nationality. Detailed characteristics and clinical information of these participants were previously described [21], including the following: (a) MMD patients presented with ischemic symptoms (either unilateral or bilateral); (b) varied Suzuki stages, with a higher number of patients at advanced stages; and (c) none of the participants had diabetes or hyperlipidemia. Data were processed using the same methodology as described in the “DNA Methylation Analysis” section.

**Table 1:** Demographics and clinical features of study subjects

Non-Asian				Asian			
	MMD	Control	*p*		MMD	Control	*p*
Number	13	7		Number	14	3	
Age at blood sampling (y) average ± SEM	33.4 ± 2.2	33.6 ± 2.6	0.96	Age at blood sampling (y) average ± SEM	42.8 ± 2.6	31.0 ± 2.6	0.059
Sex (female)	13 (100%)	7 (100%)	1	Sex (female)	14 (100%)	3 (100%)	1
RNF213 founder mutation rs112735431 (p.R4810K)	0 (0%)	0 (0%)	1	RNF213 founder mutation rs112735431 (p.R4810K)	4 (28.6%)	0 (0%)	1
Bilateral MMD	13 (100%)	NA		Bilateral MMD	13 (100%)	NA	
Disease type (symptom) (*N*, %)				Disease type (symptom) (*N*, %)			
TIA	8 (61.5%)	NA		TIA	11 (78.6%)	NA	
Headache	5 (38.5%)	NA		Headache	3 (21.4%)	NA	
Asymptomatic	0	NA		Asymptomatic	0	NA	
Angiographical disease stage (Suzuki’s stage) (*N*, %)				Angiographical disease stage (Suzuki’s stage) (*N*, %)			
2	1 (7.7%)	NA		2	1 (7.1%)	NA	
3	10 (76.9%)	NA		3	12 (85.7%)	NA	
4	2 (15.4%)	NA		4	1 (7.1%)	NA	
Hyperlipidemia	0 (0%)	0 (0%)		Hyperlipidemia	0 (0%)	0 (0%)	
Diabetes	0 (0%)	0 (0%)		Diabetes	0 (0%)	0 (0%)	

*MMD* moyamoya disease, *NA* not applicable, *RNF* ring finger protein, *SEM* standard error of mean, *TIA* transient ischemic attack

### DNA Extraction and Methylation Microarray

Whole blood samples were collected and DNA was extracted using QIAGEN AllPrep DNA/RNA Mini kit (Cat No.: 80204, Qiagen, Hilden, Germany) via QIAcube automation. NanoDrop1000 was used for DNA quantification and QC measurement. All DNA samples have 260/280 ratio > 1.75. DNA samples were then processed with bisulfide conversion using the EZ DNA Methylation Kit (Zymo Research Corporation, Irvine, CA). Genome-wide methylation profiling was performed using Infinium Methylation EPIC BeadChip Kit (Illumina Inc., San Diego, CA) that covers 850,000 human CpG probes. The EPIC methylation array was conducted at the Stanford Functional Genomics Facility, Stanford University. All samples were processed together to avoid potential batch effects.

### DNA Methylation Analysis

All DNA methylation analyses were conducted with R software (v3.6.2; R Foundation for Statistical Computing, Vienna, Austria; www.R-project.org) on Linux OS (Debian v10). IDAT files were preprocessed using the Minfi package (v1.32) [35]. Quality control involved removing low detecting probes, low-quality probes, and those at problematic locations, which include multi-mapped regions, common SNPs, and cross-reactive probes. This was completed using both built-in annotations from Minfi and publicly available databases [36]. Probes were normalized using a stratified quantile normalization method with the preprocessQuantile function from Minfi. Batch effects and cell-type heterogeneity were assessed using EpiDISH (v2.2.2) [37]. This process yielded both beta and *M*-values. Statistical analyses were conducted using *M*-values, while beta values were used for graphical representations [38]. Additional annotations, such as promoter regions and island predictions, were sourced from Bioconductor’s IlluminaHumanMethylationEPICanno.ilm10b4.hg19 package (v0.6). Single differential methylation probe (DMP) analysis was executed using Limma’s linear modeling (v3.42.2), and differential variable probe (DVP) analysis was carried out with the missMethyl package (v1.20.4) [29, 39].

### Global Distribution and Pathway Analysis in Differentially Variated CpG Probes

Difference of median coefficient of variation (CV) was compared between MMD and control groups for every significant DVP. Global distribution was mapped for DVPs according to chromosome as well as the CpG location; CpG island, open sea, shelf, and shore. Median CV was also compared between two groups regarding the CpG location. Gene Set Enrichment Analysis (GSEA) for DVPs was further analyzed for biological processes using the methylGSA package [40]. QIAGEN Ingenuity Pathway Analysis (IPA) (QIAGEN Inc., https://digitalinsights.qiagen.com/IPA) was also used to assess their biological pathways.

### Statistical Analysis

Where applicable, *p*-values were corrected for multiple testing using the Benjamini–Hochberg procedure or an equivalent method [41], such as the one implemented in the Limma package. For differential methylation analysis, we employed the Limma package, which utilizes linear modeling combined with empirical Bayes moderation for standard error estimation. To assess differences in the variance of methylation beta values across specified groups, we used the varFit function from the missMethyl package. For this function, we utilized a linear model on absolute residuals to assess differential variability. A chi-square test of independence was conducted to evaluate differences in the frequency proportions of up- and downregulated methylation levels or variability categories between differentially methylated (DMP) and differentially variated (DVP) sites, respectively. This test determines if there is a significant association between the direction of change (up/down) and the type of analysis (DMP:DVP). Additionally, for each cohort (including our Caucasian and Asian cohorts and external sets comprising males and females), we performed a binomial test to assess whether the proportion of probes showing decreased variability in MMD samples significantly deviated from the null hypothesis of equal probability (0.5) for directional changes in variability. A significant result (Bonferroni-adjusted *p* < 0.05) indicates a systematic bias toward decreased variability. To mitigate potential bias from unbalanced sample sizes, we implemented a subsampling approach for the Caucasian cohort (MMD *N* = 13, control *N* = 7). We performed 10 iterations of random subsampling, selecting 7 samples from the MMD group in each iteration to match the control group size. For each iteration, we conducted differential variability analysis between the subsampled MMD group (*N* = 7) and the full control group (*N* = 7), recording the direction of variability change for each significant probe. All measurements were conducted on distinct samples to ensure independence of observations. Additionally, we calculated the coefficient of variation (CV) as a complementary measure to screen for genome-wide variability differences.

## Results

The demographic and clinical data for MMD patients and controls are shown in Table 1. Please see the “Study Subjects” section for details on selection criteria. All participants in this study were females. RNF213 founder mutation-p. R4810K (rs112735431) was screened in all participants. In the non-Asian cohort, none of the participants carried the RNF213 founder mutation. In the Asian cohort, 28.6% of the MMD group carried the RNF213 founder mutation.

To control for potential confounders from varying cell populations, deconvolution analyses were performed to examine cell-type proportions. In our cohorts, there were no significant differences between MMD and control groups in the relative proportions of neutrophils, eosinophils, monocytes, B-cells, CD4^+^T-cells, CD8^+^T-cells, or natural killer cells in the blood samples (Online Resource 1: Supplementary Fig. 1). Batch effects were analyzed using t-distributed Stochastic Neighbor Embedding (t-SNE) to confirm there were no discrepancies between the control group and the MMD group within each cohort. In the external cohorts from China, significant differences were noted between the MMD and control groups in the relative proportions of neutrophils, B-cells, CD4^+^T-cells, and natural killer cells in the blood samples (Online Resource 1: Supplementary Fig. 2). Thus, we corrected for any significantly varying cell types in our analysis. Distinct DNA methylation patterns were observed between Asian and non-Asian cohorts, consistent with previous reports of differential methylation status associated with ethnicity [42–44]. Therefore, we proceeded to analyze non-Asian and Asian cohorts separately for all subsequent analyses.

### A Striking Difference Was Detected in Differential Variation Between MMD and Controls

**Fig. 1:**
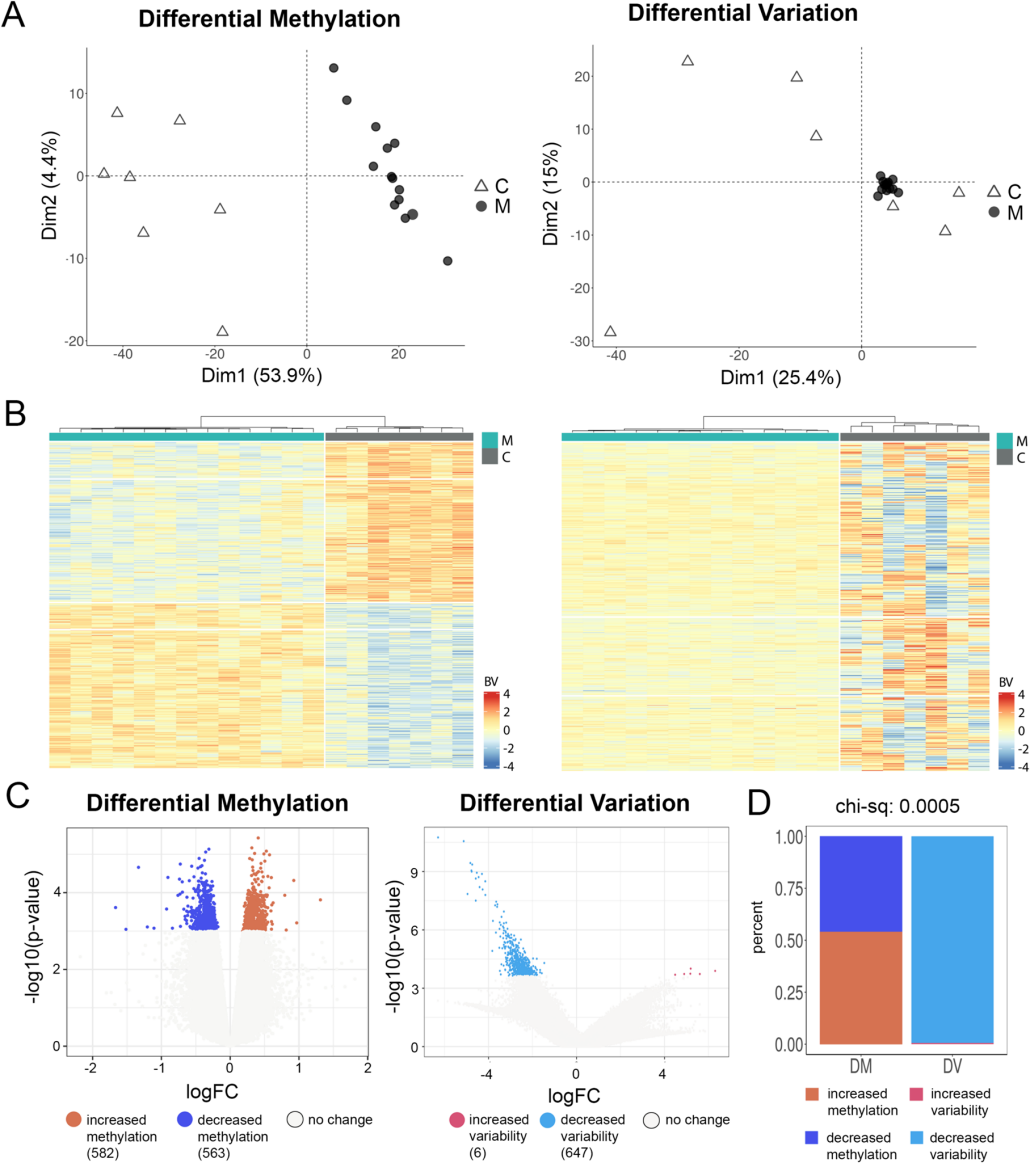
Analysis of differential methylation (DM) and differential variation (DV) in non-Asian MMD cohort. **A** Principal component analysis for differentially methylated CpG probes (DMPs, left) and differentially variated CpG probes (DVPs, right). C, healthy control, *n* = 7; M, moyamoya disease, *n* = 13; Dim, dimension. **B** Heatmaps with hierarchical cluster analysis for differential methylation (left) and differential variation (right) between groups. Red colors indicate upregulation and blue colors indicate downregulation. **C** Volcano plots of DMPs (left) and DVPs (right). Numbers of significantly methylated CpG probes (left) and variated CpG probes (right) are indicated as colored dots, DMP (left) or DVP (right) in the MMD group upregulated (up, red color) and downregulated (down, blue color) when compared to control group. **D** Comparison of the two methodological approaches of DM and DV regarding the proportion of up- and downregulated CpG probe (chi-sq, chi-square test)

In the non-Asian cohort, our analysis of differential DNA methylation analysis revealed no significant differences in mean DNA methylation between MMD and control (*p* < 0.05; FDR < 0.25). However, despite the lack of statistical significance, we proceeded with a visual examination by setting *p* < 0.001, FDR < 0.6, which resulted in 582 upregulated and 563 downregulated differentially methylated probes (DMPs) between MMD and controls (Fig. 1A–C, left). Using these DMPs, Principal component analysis (PCA), and heatmaps do show a distinct separation between groups (Fig. 1A, B, left), despite no statistical significance.

In contrast, a striking difference was detected in methylation variability between MMD and controls (*p* < 0.05; FDR < *0*.25). PCA of the differential variable probes (DVPs) showed a concentrated cluster for the MMD group and a scattered cluster for the control group (Fig. 1A, right). Heatmaps displayed a clear distinction between groups (Fig. 1B, right). The volcano plot showed only 6 DVPs were upregulated (had more variability) and 647 DVPs were downregulated (had less variability) in MMD, when compared to the control group (Fig. 1C, right).

**Fig. 2:**
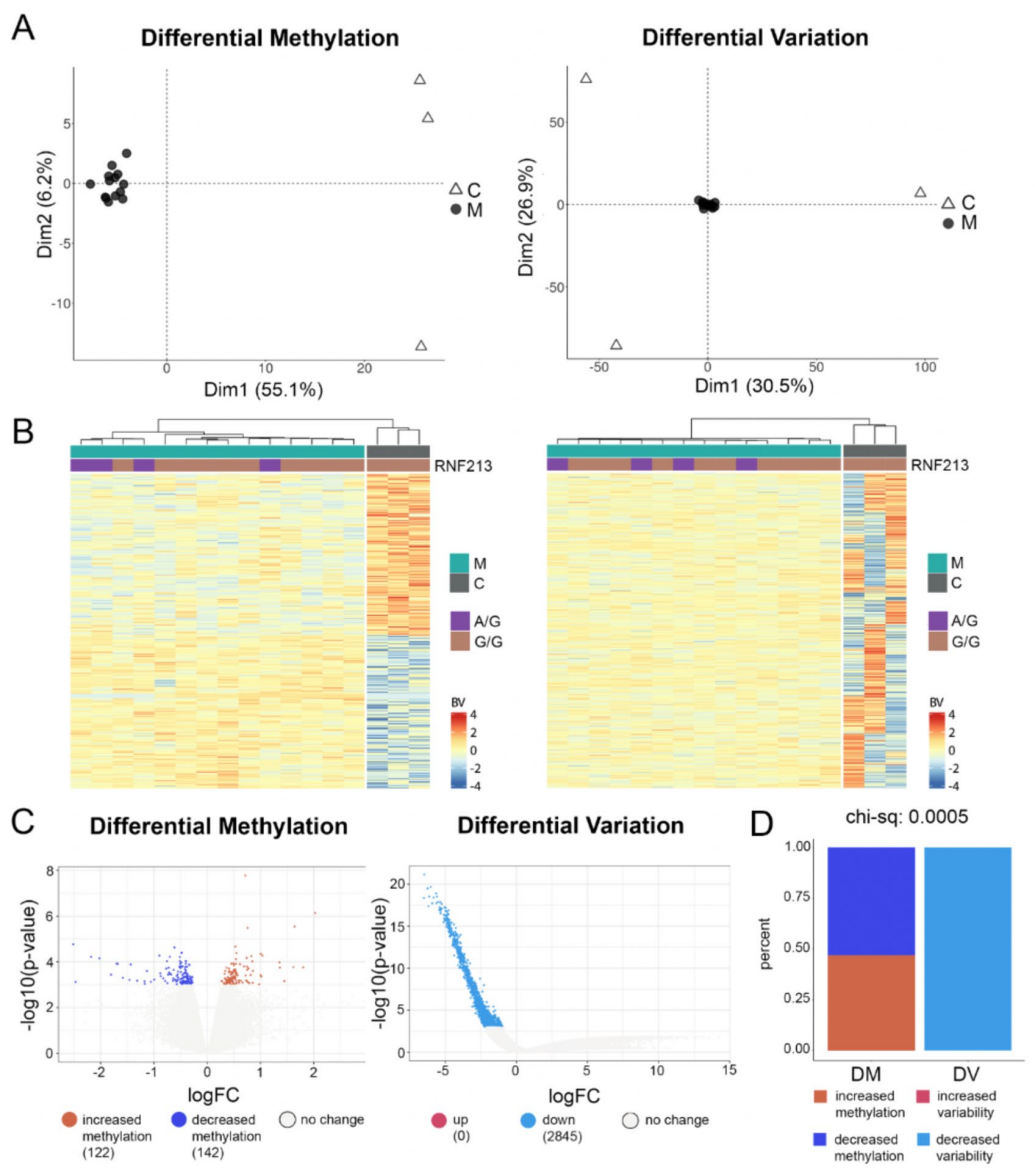
Analysis of differential methylation (DM) and differential variation (DV) in Asian MMD cohort. **A** Principal component analysis for differentially methylated CpG probes (DMPs, left) and differentially variated CpG probes (DVPs, right). C, healthy control, n=3; M, moyamoya disease, n=14; Dim, dimension. **B** Heatmaps with hierarchical cluster analysis for differential methylation (left) and differential methylation (right) between groups. Red colors indicate upregulation and blue colors indicate downregulation. Purple color indicates RNF213 A/G founder mutation; brown color indicates RNF213 wildtype. **C** Volcano plots of DMPs (left) and DVPs (right). Numbers of significantly methylated CpG probes (left) and variated CpG probes (right) are indicated as colored dots, DMP (left) or DVP (right) in the MMD group upregulated (up, red color) and downregulated (down, blue color) when compared to control group. **D** Comparison of the two methodological approaches of DM and DV regarding the proportion of up- and downregulated CpG probe (chi-sq, chi-square test)

In the Asian cohort, there were also no significant differences in mean DNA methylation between MMD and control (*p* < 0.05; FDR < 0.25). However, similar to the non-Asian group, we proceeded with a visual examination by setting *p* < 0.001 without FDR correction, which resulted in 122 upregulated and 142 downregulated DMPs (Fig. 2, left). Supervised PCA and heatmaps (Fig. 2A, B, left) also displayed distinct clusters between groups. Similar to the non-Asian cohort, we observed a difference in differential variability between MMD and control groups (*p* < 0.05; FDR < 0.25) (Fig. 2). PCA of the DVPs showed a concentrated cluster for the MMD group and a scattered cluster for the control group (Fig. 2A, right). Heatmaps displayed a clear distinction between groups (Fig. 2B, right). In our Asian cohort, we identified 4 MMD patients with the founder mutation RNF213. Hierarchical cluster analysis showed that these patients did not form a specific cluster and exhibited similar low variability to other MMD patients. Volcano plot showed all significant DVPs were downregulated in MMD (2845 DVPs), when compared to the control group (Fig. 2C, right). There were zero probes that had a significant increase in variability in the MMD group. These findings demonstrated a significantly reduced methylation variability in MMD, irrespective of their racial background.

Both the non-Asian and Asian MMD cohorts demonstrate a significant skew in DVP, favoring less variability; six increased variability versus 647 decreased variability in the non-Asian MMD group, and zero increased variability versus 2845 decreased variability in the Asian MMD cohort, respectively. This is in contrast to differential methylation, which was evenly distributed between increase and decrease in methylation in both racial MMD groups. We utilized a chi-square test to quantitatively assess this disparity between differential variability and methylation changes, revealing a significant imbalance with a *p*-value of 0.0005 for both cohorts (Figs. 1D and 2D).

### External Cohort Validation in Differential Variation Between MMD and Controls

**Fig. 3:**
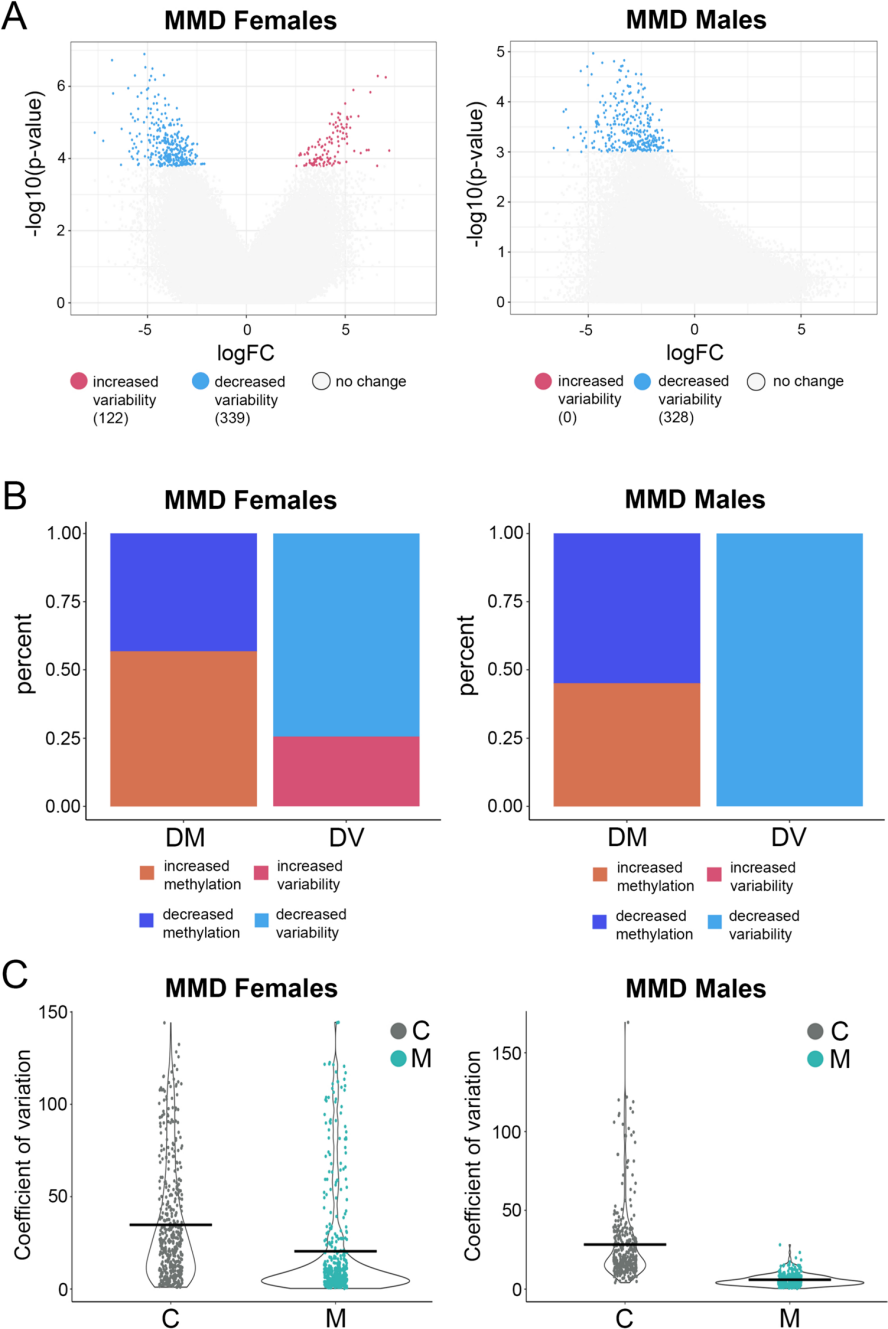
Differential methylation variability analysis in external MMD cohort. **A** Volcano plots of differential variated probes (DVPs) in MMD females (left) and males (right). DVPs are shown as colored dots, increased variability (red color) and decreased variability in MMD (blue color) when compared to the control group. **B** Comparison of the two methodological approaches of DM and DV regarding the proportion of increased and decreased methylated or variated CpG probe (chi-square test, *p* = 0.0005). **C** Scatter plot showing the coefficient of variation (CV) of each DVP in healthy control (C) and MMD (M) group in females (left) and males (right). This external cohort includes10 MMD patients (*n* = 5 males, *n* = 5 females) and 10 healthy controls (*n* = 5 males, *n* = 5 females)

To validate the findings we observed in our cohorts, we analyzed DNA methylation variability in an external cohort (Fig. 3). This external cohort consists of 10 MMD patients (*n* = 5 males, *n* = 5 females) and 10 healthy controls (*n* = 5 males, *n* = 5 females). Volcano plot showed 122 DVPs significantly upregulated and 339 DVPs significantly downregulated (had less variability) in MMD females, when compared to the control group (*p* < 0.05; FDR < 0.25) (Fig. 3A, left). Although no DVPs met our significance cutoffs in the male cohort, removing the FDR revealed a similar trend, with all 328 DVPs downregulated in MMD males (*p* < 0.001, no FDR applied), mirroring the changes observed in the female group (Fig. 3A, right). Similar to our previous findings, both female and male MMD cohorts demonstrate a significant skew in DVP with MMD having more probes with lower variability. Likewise, with differential methylation, the ratio between upregulated and downregulated probes was balanced. Moreover, to analyze the disparity between the two types of methylation changes in this external cohort, we conducted a chi-square test. This analysis similarly revealed significant differences, yielding a *p*-value of 0.0005 for both sexes (Fig. 3). The data from this external cohort align with our findings, confirming that MMD patients (both sexes) display reduced methylation variability.

To ensure the reliability of our findings and further validate the observed decrease in variability in MMD patients, we conducted additional binomial tests for each cohort. This test assessed whether the proportion of probes showing decreased variability in MMD samples significantly deviated from the null hypothesis of equal probability (0.5) for changes in variability. A significant result (*p* < 0.05) indicates a systematic bias toward decreased variability. As expected, all cohorts showed a significant bias toward the decrease in variability in MMD (Supplementary Table 1). Furthermore, we recognize that low sample sizes, coupled with imbalance between the MMD and control groups as in the case with the Caucasian cohort (MMD *N* = 13, control *N* = 7), could potentially bias the results, particularly in differential variability analysis. Thus, to mitigate this potential bias, we employed a subsampling approach coupled with multiple testing correction (Supplementary Table 2): (1) subsampling—we performed 10 iterations of random subsampling, wherein 7 samples were randomly selected from the experimental group for each iteration to match the control group size; (2) iterative analysis. In each iteration, differential variability analysis was conducted between the subsampled experimental group (*N* = 7) and the full control group (*N* = 7); (3) direction assessment—the direction of variability change (increased or decreased) for each significant probe was recorded across iterations; (4) statistical testing—a binomial test was applied to assess the bias toward decreased variability in each iteration; (5) multiple testing correction—to account for multiple comparisons, the resulting *p*-values from each iteration were adjusted using Bonferroni correction.

### Global Distribution of Differentially Variated CpG Probes

**Fig. 4:**
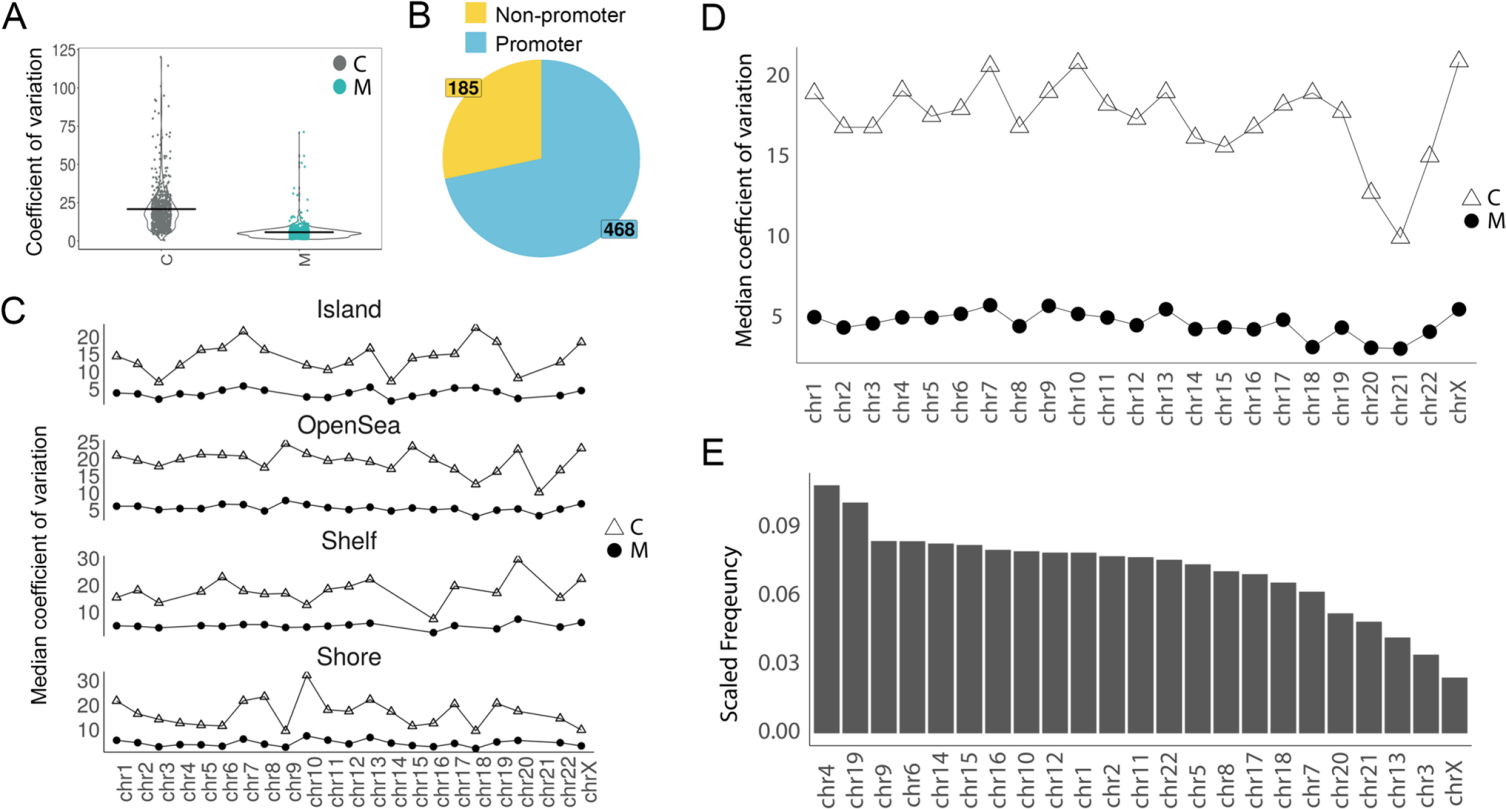
Global distribution of differentially variated CpG probes (DVPs) in non-Asian MMD cohort. **A** Scatter plot showing the coefficient of variation (CV) of each significant DVP in healthy control (C) (*n* = 7) and MMD (M) (*n* = 13) group. **B** Pie chart shows proportion of significant DVPs associated with promoter. Yellow = non-promoter associated; blue = promoter associated. DVPs associated with promoter were defined by whether the probes land on areas with the following annotations: TSS, 5′UTR, 1st Exon, and promoter. **C** Graph depicts median coefficient of variation (CV) for all significant DVPs for each chromosome by group (C vs M), in different promotor regions (island, open sea, shelf, and shore). **D** Graph depicts median coefficient of variation (CV) for all significant probes for each chromosome by group (C vs M). **E** Frequency of significant DVPs across the genome. *Y*-axis is frequency of significant DVPs scaled by total QC-filtered probes represented in the EPIC platform for each chromosome. *X*-axis is ranked by highest to lowest total scaled frequency

Next, we directed our subsequent analysis toward these differentially variable CpG probes (DVPs). One of our primary inquiries was to investigate the location and distribution of these DVPs at a genomic level. In the non-Asian cohort, a comparison of the median coefficient of variation (CV) of the DVPs between the control and MMD groups showed overall low variability in methylation status in the MMD patients (Fig. 4A). Regional analysis of the 653 DVPs showed that 185 DVPs were localized in non-promoter-associated regions, and 468 DVPs were localized in promoter-associated regions (Fig. 4B). Figure 4C further shows the variation comparison of the DVPs across different regions in the chromosomes, including island, open sea, shelf and shore. The variance in the control is consistently higher than the MMD group, irrespective of chromosomes (Fig. 4D). Frequency histogram analysis of these DVPs showed that there is no significant trend across chromosomes (Fig. 4E).

**Fig. 5:**
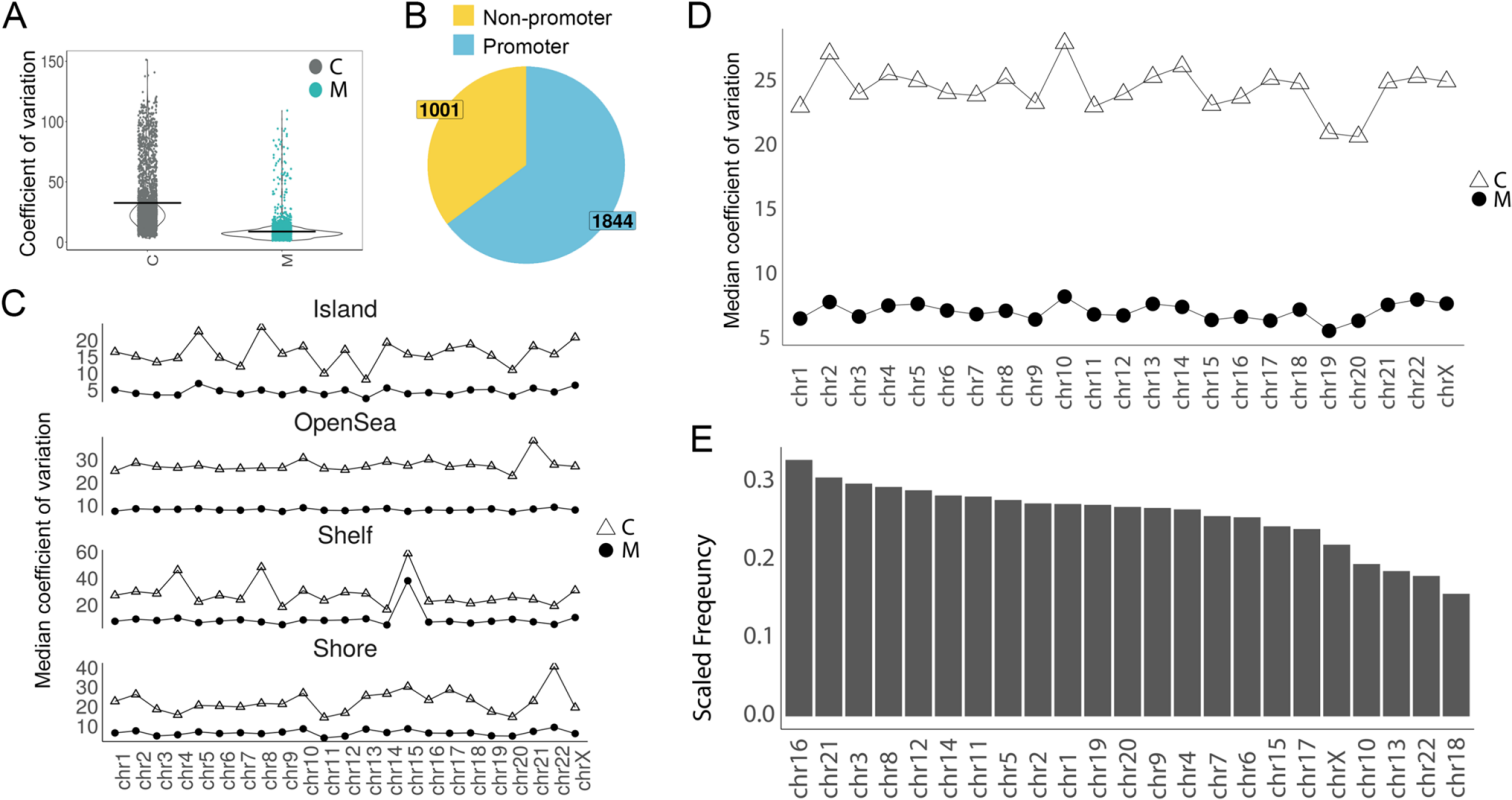
Global distribution of differentially variated CpG probes (DVPs) in Asian MMD cohort. **A** Scatter plot showing the coefficient of variation (CV) of each significant DVP in healthy control (C) (n=3) and MMD (M) (n=14) group. **B** Pie chart shows proportion of significant DVPs associated with promoter. Yellow = non-promoter associated; blue = promoter associated. DVPs associated with promoter were defined by whether the probes land on areas with the following annotations: TSS, 5′UTR, 1st Exon, and promoter. **C** Graph depicts median coefficient of variation (CV) for all significant probes for each chromosome by group (C vs M), in different promotor regions (island, open sea, shelf, and shore). **D** Graph depicts median coefficient of variation (CV) for all significant probes for each chromosome by group (C vs M). **E** Frequency of significant DVPs across the genome. *Y*-axis is frequency of significant DVPs scaled by total QC-filtered probes represented in the EPIC platform for each chromosome. *X*-axis is ranked by highest to lowest total scaled frequency

The Asian cohort exhibited very similar patterns in their global distribution (Fig. 5). The variance of DVPs between control and MMD group also showed overall low variability in methylation status in the MMD patients (Fig. 5A). This global reduced low methylation variability was also confirmed in the external Asian cohort in both female and male MMD patients (Fig. 3C). Regional analysis of the 2845 significant DVPs showed that 1001 DVPs were localized in non-promoter-associated regions and 1844 DVPs were localized in promoter-associated regions (Fig. 5B). Figure 5C shows the comparison of the DVPs across different regions in the chromosomes, including island, open sea, shelf, and shore. The variance in the control is consistently higher than that in the MMD group across chromosomes (Fig. 5D). Similar to the non-Asian cohort, frequency histogram analysis indicated that there are no hot spots of these DVPs on specific chromosomes (Fig. 5E).

### Pathway Analysis of the Differentially Variated CpG Probes

**Fig. 6:**
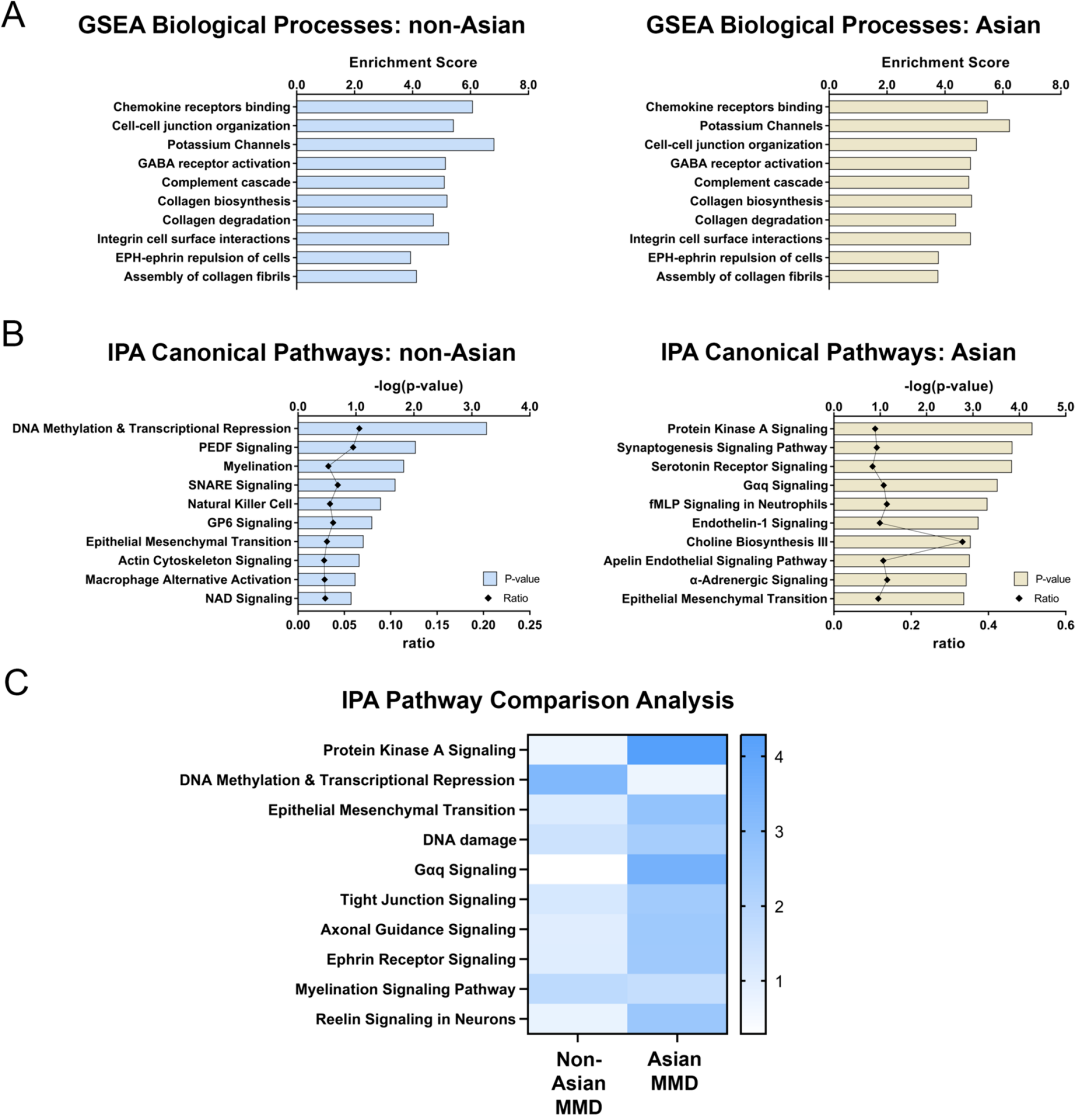
Biological processes and pathway analysis of genes associated with the differentially variated CpG probes (DVPs). **A** Top 10 biological pathways in non-Asian (left) and Asian cohort (right) using GSEA biological processes with the Reactome database. Each pathway is ranked by enrichment scores. **B** Top 10 IPA canonical pathways in non-Asian (left) and Asian cohort (right). Each pathway is ranked by − log(*p*-value), which represents the significance of the association between the genes in a specific pathway. Ratio is plotted to show the fraction of genes from our dataset that mapped to a particular pathway. **C** IPA comparison analysis between non-Asian and Asian MMD cohorts. Blue color represents − log(*p*-value). Darker blue color equals higher significance. Non-Asian cohort: *n* = 7 for control and *n* = 13 for MMD; Asian cohort: *n* = 3 for control and *n* = 14 for MMD

The genes where these DVPs were localized were identified and further analyzed using various pathway analysis tools to determine associated biological processes and molecular functions. Figure 6A shows similar top 10 biological processes from Gene Set Enrichment Analysis (GSEA) for both cohorts (left: non-Asian cohort, right: Asian cohort), including chemokine receptors binding, cell–cell junction organization, potassium channels, GABA receptor activation, complement cascade, collagen biosynthesis, collagen degradation, integrin cell surface interactions, EPH-ephrin repulsion of cells, and assembly of collagen fibrils. We also performed ingenuity pathway analysis to further investigate the biological signaling pathways these genes are involved in each cohort (Fig. 6B). In the non-Asian cohort, some of the top biological signaling pathways include DNA methylation and transcriptional repression, pigment epithelium-derived factor (PEDF) signaling, natural killer cell signaling, epithelial mesenchymal transition, and actin cytoskeleton signaling. In the Asian cohort, some of the top biological signaling pathways include protein kinase A signaling, endothelin-1 signaling, choline biosynthesis, and epithelial mesenchymal transition. To identify key pathways that are common in both cohorts, we performed a pathway comparison analysis (Fig. 6C) between non-Asian MMD and Asian MMD cohorts. The common pathways present in both cohorts include protein kinase A signaling, DNA methylation and transcriptional repression, epithelial mesenchymal transition, DNA damage, Gαq signaling, tight junction signaling, axonal guidance, ephrin receptor signaling, myelination, and reelin signaling.

### Common Key Genes in Non-Asian and Asian MMD

**Fig. 7:**
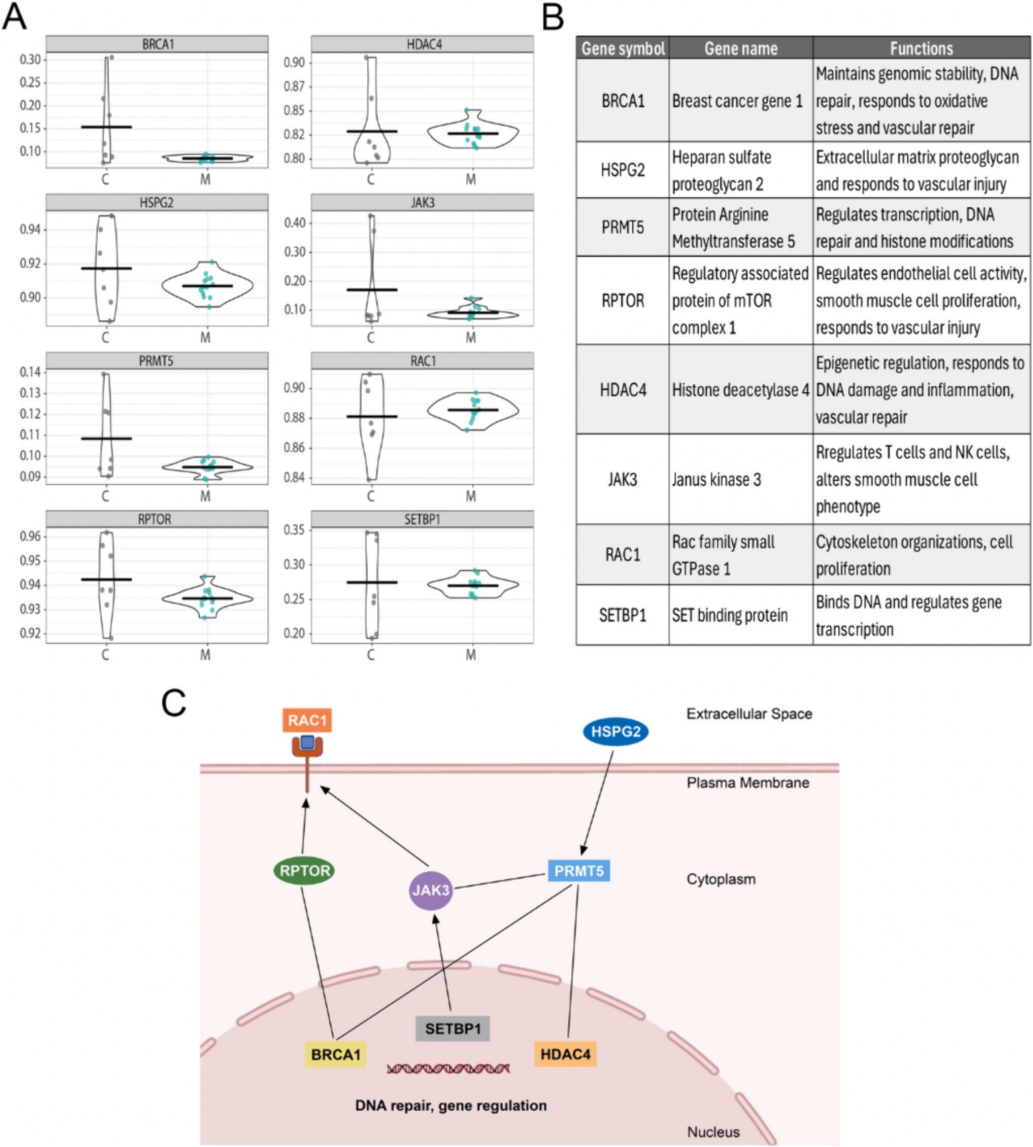
Common genes between non-Asian and Asian MMD. **A** Scattered violin plots of key genes common in both MMD cohorts (BRCA1, HSPG2, PRMT5, RPTOR, HDAC4, JAK3, RAC1, and SETBP1). *Y*-axis represents beta value, which is a measure of DNA methylation levels at a specific CpG site. Non-Asian cohort: *n* = 7 for control and *n* = 13 for MMD. **B** Table lists the key common gene symbols, names, and their functions in relation to vascular injury, DNA repair/transcription, and inflammation. **C** The diagram on the left illustrates the signaling pathways of these key common genes. Disruption of these signaling pathways may result in the dysregulation of DNA repair, methylation, and transcriptional regulation, altering their response to vascular injury and potentially contributing to MMD pathogenesis. Created with BioRender.com

To identify pivotal genes potentially linked to MMD pathology, we investigated common genes across both non-Asian and Asian MMD groups. Venn diagram analysis revealed 46 intersecting genes between these two cohorts. We then chose eight genes that were prominent in the key common pathways, as illustrated in Fig. 6C. These pathways include DNA methylation and transcription, DNA repair, cytoskeleton remodeling, and natural killer cell signaling, as well as cellular growth and migration. The DVPs associated with these selected eight genes are depicted in Fig. 7A, highlighting the contrasting variability between the control and MMD groups. These eight genes (Fig. 7B) include the Rac family small GTPase 1 (RAC1), regulatory associated protein of mTOR complex 1 (RPTOR), Janus kinase 3 (JAK3), protein arginine methyltransferase 5 (PRMT5), heparan sulfate proteoglycan 2 (HSPG2), breast cancer gene 1 (BRAC1), SET binding protein (SETBP1), and histone deacetylase 4 (HDAC4).

## Discussion

Our findings revealed remarkable differences in differential methylation variability among MMD patients compared to controls. Genome-wide DNA methylation analysis showed a strikingly low methylation variability in both MMD cohorts (non-Asian and Asian), when compared to matched controls (Figs. 1 and 2). This finding was further validated in an external Asian cohort, in both female and male MMD patients (Fig. 3). Moreover, these significantly muted probes appear across the chromosomes without any discernable patterns, suggesting a broad decrease in the entire genome (Figs. 4 and 5). GSEA of the genes associated with these DVPs showed similar biological processes in both MMD cohorts, including immune-related, cellular interactions and cell migration signaling (Fig. 6A). IPA revealed various top biological pathways for each MMD cohort (Fig. 6B), including DNA methylation and transcription, DNA repair, natural killer cell signaling, and cytoskeleton remodeling, as well as cell growth and migration signaling (Fig. 6B, C). We further investigated key genes (Fig. 7B) potentially linked to MMD pathology and identified 46 intersecting genes between these non-Asian and Asian cohorts. Some of these genes were highlighted in the key common pathways illustrated in Fig. 6C. Figure 7A comprises variability plots that depict the remarkably low variability of the DVPs in the MMD group, in both cohorts. Taken together, our findings reveal significantly low methylation variability in MMD patients, which could hinder their ability to adapt to environmental changes, impairing various biological processes and potentially contributing to MMD pathology.

Accumulating reports highlight epigenetic changes in various diseases, such as cerebrovascular and neurological diseases [22, 25, 26, 45]. For example, in cerebral ischemic stroke, an epigenome-wide association study identified a differentially methylated region in the promoter of the humanin gene that may be involved in the protection against cognitive impairment, stroke, and inflammatory response [27]. Similarly, DNA methylation changes have been reported in several neurological diseases, such as multiple sclerosis [46] and Parkinson’s disease [47]. However, most studies focused on mean DNA methylation, where differences are often minimal, making it difficult to draw meaningful biological conclusions. In contrast, variability in DNA methylation is more substantial and increasingly studied for its potential impact on disease pathology and susceptibility. Several studies found that increased DNA methylation variability in multiple genes was associated with cancers [32], type 1 diabetes [31], aging [48], and rheumatoid arthritis [30]. Huo et al. also reported DNA methylation variability profiles in Alzheimer’s disease and found that most of the DVPs did not overlap with DMPs [49], indicating that DVPs and DMPs might capture different sets of genes associated with disease pathology.

In MMD, the role of epigenetics such as DNA methylation is largely unknown. To date, we are not aware of any study that has examined DNA methylation variability in moyamoya disease. There is one study that reported changes in mean DNA methylation in an Asian MMD cohort (Chinese) that included both sexes [21]. Interestingly, their mean methylation data revealed several genes that may be involved in vascular occlusion in MMD, such as SOX6 and RBM33. This original study did not explore methylation variability in their cohort, and upon our request, the authors graciously shared their raw data. This allowed us to analyze their data to validate our findings as an external cohort. Here, we showed similar results, with significantly lower methylation variability in MMD females and a strong trend of low methylation variability in MMD males (Fig. 3). However, these results were not as robust as those observed in our cohorts, most likely due to a number of factors such as a smaller sample size, variations in blood collection sample processing methods, and differences among subpopulations of Han Chinese. In our cohorts, the DVPs were located on genes involved in DNA methylation and transcription, DNA repair, natural killer cell signaling, cytoskeleton remodeling, cell growth, and migration signaling. Intersectional analysis between non-Asian and Asian cohorts revealed 46 common genes. Of these, we highlighted eight genes that were highly represented in the pathway analysis (RAC1, RPTOR, JAK3, PRMT5, HSPG2, BRAC1, SETBP1, and HDAC4) (Fig. 7). RAC1, a GTPase that regulates cytoskeleton organization, transcription, and cell proliferation, influences RPTOR/mTOR signaling during cellular stress, which is crucial for vascular functions such as endothelial cell activity, smooth muscle cell proliferation, and the response to vascular injury [50]. JAK3 regulates the development of lymphocytes (T-cells and natural killer cells) [51, 52], and its deficiency enhances endothelial recovery postinjury and alters smooth muscle cells from synthetic to contractile phenotypes [52]. PRMT5 controls transcription through protein methylation [53, 54] and enhances DNA repair efficiency by regulating histone modifications and repair proteins [55]. SETBP1 binds DNA to increase gene expression; dysregulation of this binding leads to altered transcriptomes [56]. HSPG2, an extracellular matrix proteoglycan, plays a key role in vascular response to injury [57, 58]. Together, disruption of signaling pathways mediated by these genes may contribute to MMD pathology.

To our knowledge, this is the first study to explore methylation variability in MMD, and it reports the first instance of low methylation variability in any disease, contrasting with higher variability observed in other diseases such as cancer [32], type 1 diabetes [31], aging [48], Alzheimer’s disease [49], and rheumatoid arthritis [30]. High methylation variability observed in cancer may be due to tumor heterogeneity and genomic instability of tumor cells [32]. Similarly, in autoimmune diseases such as rheumatoid arthritis and type 1 diabetes, increased variability in DNA methylation has been associated with the immune system’s dynamic response to ongoing inflammation and immune challenges [30, 31]. Increased methylation variability could be an adaptive mechanism, allowing cells to respond to environmental changes. In contrast, low methylation variability, as observed in MMD, represents a unique and significant finding, suggesting a distinct pathophysiological mechanism where the epigenetic landscape is tightly controlled. This rigidity implies tighter regulation of gene expressions, potentially limiting the cells’ ability to adapt to changing environmental conditions, such as hemodynamic stress or blood oxygen saturation levels, which are particularly relevant in cerebrovascular diseases like MMD. The onset or progression of MMD may involve factors beyond genetics, regardless of the presence or absence of an RNF213 gene mutation. These factors could include immune responses to viral infections, autoimmune system irregularities, and hormonal imbalances. The low methylation variability in MMD could suggest a more stable and uniform epigenetic environment, possibly resulting from the chronic nature of the disease and the ongoing vascular stress. This stability might hinder the cells’ adaptability to external changes, potentially leading to poorer outcomes. Considering that MMD typically manifests in the intracranial internal carotid arteries [59], the low methylation variability observed in MMD could limit the ability to adapt to shifts in hemodynamic stress or blood oxygen saturation levels at these arteries and affect the maintenance of vascular homeostasis. This could contribute to abnormal intimal thickening in the internal carotid arteries, exacerbating the disease by promoting the narrowing of the arteries and leading to ischemic symptoms. In this study we chose to analyze circulating blood cells due to the non-invasive nature of blood sampling and the practical challenges of obtaining intracranial tissue. Blood-based epigenetic changes can provide valuable insights into systemic biological processes and the broader gene-environment interactions relevant to MMD. Circulating blood represents the immediate environment for vascular tissues, making it crucial to study potential epigenetic changes that could influence disease pathology. Additionally, emerging studies have indicated that immune responses might play a significant role in the development of MMD [19, 20, 60, 61]. By examining circulating blood cells, we can capture these systemic immune responses and their potential impact on the disease. Future research could correlate these blood-based findings with intracranial tissue data to further validate and expand our understanding of MMD pathology.

The limitation of our study includes a small cohort size and largely focused on female participants, which may introduce sex-specific biases and limit the generalizability of the findings. Future research will involve larger, mixed-sex cohorts to validate and expand upon our findings. In addition, cohort-specific biases, such as ethnic and genetic background, may have influenced our results, and future studies will aim to include larger diverse populations to account for these potential variables. Furthermore, our research was limited to the adult populations. Since MMD occurs in both children and adults, expanding the scope to include pediatric cases will be crucial in developing a more comprehensive understanding of MMD’s epigenetic landscape across all age groups.

Our findings revealed significant differences in methylation variability between MMD patients and healthy control across both Asian and non-Asian MMD cohorts. The low methylation variability observed in MMD is a unique and significant finding, marking the first report of low methylation variability in any disease. The DVPs with low methylation variability are located in genes regulating DNA methylation and transcription, DNA repair, natural killer cell signaling, cytoskeleton remodeling, cellular growth, and migration. Our study highlights the importance of epigenetic variability as an emerging biologically relevant feature in human disease and encourages further investigation of epigenetic variability in other cerebrovascular diseases, neurological diseases, and psychiatric diseases.

## Supplementary Information

Below is the link to the electronic supplementary material.

Supplementary file1 (DOCX 1416 KB)

## Data Availability

The datasets generated/analyzed in the current study are not publicly available due to patient privacy, but are available from the corresponding author on reasonable request.

## References

[1] Weinberg DG, Arnaout OM, Rahme RJ, et al. Moyamoya disease: a review of histopathology, biochemistry, and genetics. Neurosurg Focus. 2011;30:E20. 21631222 10.3171/2011.3.FOCUS1151

[2] Suzuki J, Takaku A. Cerebrovascular “moyamoya” disease. Disease showing abnormal net-like vessels in base of brain. Arch Neurol. 1969;20:288–99. 5775283 10.1001/archneur.1969.00480090076012

[3] Weinberg DG, Arnaout OM, Rahme RJ, et al. Moyamoya disease: a review of histopathology, biochemistry, and genetics. Neurosurg Focus. 2011;30:20. 10.3171/2011.3.FOCUS1151 21631222

[4] Kuriyama S, Kusaka Y, Fujimura M, et al. Prevalence and clinicoepidemiological features of moyamoya disease in Japan: findings from a nationwide epidemiological survey. Stroke. 2008;39:42–7. 18048855 10.1161/STROKEAHA.107.490714

[5] Kamada F, Aoki Y, Narisawa A, et al. A genome-wide association study identifies RNF213 as the first Moyamoya disease gene. J Hum Genet. 2011;56:34–40. 21048783 10.1038/jhg.2010.132

[6] Liu W, Morito D, Takashima S, et al. Identification of RNF213 as a susceptibility gene for moyamoya disease and its possible role in vascular development. PLoS One. 2011;6(7):22542. 10.1371/journal.pone.0022542 PMC3140517 21799892

[7] Zhang H, Zheng L, Feng L. Epidemiology, diagnosis and treatment of moyamoya disease (Review). Exp Ther Med. 2019;17(3):1977–84. 30867689 10.3892/etm.2019.7198 PMC6395994

[8] Kainth D, Chaudhry SA, Kainth H, et al. Epidemiological and clinical features of moyamoya disease in the USA. Neuroepidemiology. 2013;40:282–7. 23445954 10.1159/000345957

[9] Kundishora AJ, Peters ST, Pinard A, et al. DIAPH1 variants in non–East Asian Patients with sporadic moyamoya disease. JAMA Neurol. 2021;78:993–1003. 34125151 10.1001/jamaneurol.2021.1681 PMC8204259

[10] Guey S, Tournier-Lasserve E, Hervé D. Kossorotoff M Moyamoya disease and syndromes: from genetics to clinical management. Appl Clin Genet. 2015;8:49–68. 25733922 10.2147/TACG.S42772 PMC4337618

[11] Mertens R, Graupera M, Gerhardt H, et al. The genetic basis of moyamoya disease. Transl Stroke Res. 2022;13:25–45. 34529262 10.1007/s12975-021-00940-2 PMC8766392

[12] Scott RM, Smith ER. Moyamoya disease and moyamoya syndrome. N Engl J Med. 2009;360:1226–37. 19297575 10.1056/NEJMra0804622

[13] Kim SK, Il Yoo J, Cho BK, et al. Elevation of CRABP-I in the cerebrospinal fluid of patients with moyamoya disease. Stroke. 2003;34:2835–41. 14605320 10.1161/01.STR.0000100159.43123.D7

[14] Kang HS, Kim JH, Phi JH, et al. Plasma matrix metalloproteinases, cytokines and angiogenic factors in moyamoya disease. J Neurol Neurosurg Psychiatry. 2010;81:673–8. 19965844 10.1136/jnnp.2009.191817

[15] Fujimura M, Watanabe M, Narisawa A, et al. Increased expression of serum matrix metalloproteinase-9 in patients with moyamoya disease. Surg Neurol. 2009;72:476–80. 19147196 10.1016/j.surneu.2008.10.009

[16] Abhinav K, Lee AG, Pendharkar AV, et al. Comprehensive profiling of secreted factors in the cerebrospinal fluid of moyamoya disease patients. Transl Stroke Res. 2023;15:399–408. 36745304 10.1007/s12975-023-01135-7 PMC10891229

[17] Hamauchi S, Shichinohe H, Uchino H, et al. Cellular functions and gene and protein expression profiles in endothelial cells derived from moyamoya disease-specific iPS cells. PLoS One. 2016;11:e0163561. 27662211 10.1371/journal.pone.0163561 PMC5035048

[18] Kang HS, Moon YJ, Kim YY, et al. Smooth-muscle progenitor cells isolated from patients with moyamoya disease: novel experimental cell model. J Neurosurg. 2014;120:415–25. 24160477 10.3171/2013.9.JNS131000

[19] Peng X, Zhang Z, Ye D, et al. Gene dysregulation in peripheral blood of moyamoya disease and comparison with other vascular disorders. PLoS One. 2019;14(8):e0221911. 31532776 10.1371/journal.pone.0221811 PMC6750579

[20] Tang Q, Li W, Huang J, et al. Single-cell sequencing analysis of peripheral blood in patients with moyamoya disease. Orphanet J Rare Dis. 2023;18:1–12. 37400835 10.1186/s13023-023-02781-8 PMC10318666

[21] He S, Ye X, Duan R, et al. Epigenome-wide association study reveals differential methylation sites and association of gene expression regulation with ischemic moyamoya disease in adults. Oxid Med Cell Longev. 2022;2022:7192060. 35368875 10.1155/2022/7192060 PMC8970806

[22] Robertson KD. DNA methylation and human disease. Nat Rev Genet. 2005;6:597–610. 16136652 10.1038/nrg1655

[23] Klutstein M, Nejman D, Greenfield R, Cedar H. DNA methylation in cancer and aging. Cancer Res. 2016;76:3446–50. 27256564 10.1158/0008-5472.CAN-15-3278

[24] Toghill BJ, Saratzis A, Harrison SC, et al. The potential role of DNA methylation in the pathogenesis of abdominal aortic aneurysm. Atherosclerosis. 2015;241:121–9. 25974102 10.1016/j.atherosclerosis.2015.05.001

[25] Urdinguio RG, Sanchez-Mut JV, Esteller M. Epigenetic mechanisms in neurological diseases: genes, syndromes, and therapies. Lancet Neurol. 2009;8:1056–72. 19833297 10.1016/S1474-4422(09)70262-5

[26] Martínez-Iglesias O, Carrera I, Carril JC, et al. DNA methylation in neurodegenerative and cerebrovascular disorders. Int J Mol Sci. 2020;21:2220. 32210102 10.3390/ijms21062220 PMC7139499

[27] Shen Y, Peng C, Bai Q, et al. Epigenome-wide association study indicates hypomethylation of MTRNR2L8 in large-artery atherosclerosis stroke. Stroke. 2019;50:1330–8. 31084332 10.1161/STROKEAHA.118.023436

[28] Palumbo D, Affinito O, Monticelli A, Cocozza S. DNA methylation variability among individuals is related to CpGs cluster density and evolutionary signatures. BMC Genomics. 2018;19:229. 29606093 10.1186/s12864-018-4618-9 PMC5880022

[29] Phipson B, Oshlack A. DiffVar: a new method for detecting differential variability with application to methylation in cancer and aging. Genome Biol. 2014;15:465. 25245051 10.1186/s13059-014-0465-4 PMC4210618

[30] Webster AP, Plant D, Ecker S, et al. Increased DNA methylation variability in rheumatoid arthritis-discordant monozygotic twins. Genome Med. 2018;10:64. 30176915 10.1186/s13073-018-0575-9 PMC6122744

[31] Paul DS, Teschendorff AE, Dang MAN, et al. Increased DNA methylation variability in type 1 diabetes across three immune effector cell types. Nat Commun. 2016;7:13555. 27898055 10.1038/ncomms13555 PMC5141286

[32] Hansen KD, Timp W, Bravo HC, et al. Increased methylation variation in epigenetic domains across cancer types. Nat Genet. 2011;43:768. 21706001 10.1038/ng.865 PMC3145050

[33] Teo M, Abhinav K, Bell-Stephens TE, et al. Short- and long-term outcomes of moyamoya patients post-revascularization. J Neurosurg. 2022;138:1374–84. 36272120 10.3171/2022.8.JNS22336

[34] Kuroda S, Fujimura M, Takahashi J, et al. Diagnostic criteria for moyamoya disease-2021 revised version. Neurol Med Chir. 2022;62:307–12. 10.2176/jns-nmc.2022-0072 PMC9357455 35613882

[35] Aryee MJ, Jaffe AE, Corrada-Bravo H, et al. Minfi: a flexible and comprehensive Bioconductor package for the analysis of Infinium DNA methylation microarrays. Bioinformatics. 2014;30:1363–9. 24478339 10.1093/bioinformatics/btu049 PMC4016708

[36] Benton MC Illumina450K_filtering: a collection of resources to filter Illumina 450k and EPIC methylation arrays

[37] Zheng SC, Breeze CE, Beck S, Teschendorff AE. Identification of differentially methylated cell types in epigenome-wide association studies. Nat Methods. 2018;15:1059–66. 30504870 10.1038/s41592-018-0213-x PMC6277016

[38] Du P, Zhang X, Huang CC, et al. Comparison of Beta-value and M-value methods for quantifying methylation levels by microarray analysis. BMC Bioinform. 2010;11:587. 10.1186/1471-2105-11-587 PMC3012676 21118553

[39] Ritchie ME, Phipson B, Wu D, et al. limma powers differential expression analyses for RNA-sequencing and microarray studies. Nucleic Acids Res. 2015;43:e47. 25605792 10.1093/nar/gkv007 PMC4402510

[40] Ren X, Kuan PF. methylGSA: a Bioconductor package and Shiny app for DNA methylation data length bias adjustment in gene set testing. Bioinformatics. 2019;35(11):1958–9. 10.1093/bioinformatics/bty892. 30346483 10.1093/bioinformatics/bty892

[41] Benjamini Y, Hochberg Y. Controlling the False discovery rate: a practical and powerful approach to multiple testing. J R Stat Soc Ser B. 1995;57:289–300.

[42] Song MA, Brasky TM, Marian C, et al. Racial differences in genome-wide methylation profiling and gene expression in breast tissues from healthy women. Epigenetics. 2015;10:1177–87. 26680018 10.1080/15592294.2015.1121362 PMC4844220

[43] Adkins RM, Krushkal J, Tylavsky FA, Thomas F. Racial differences in gene-specific DNA methylation levels are present at birth. Birth Defects Res A Clin Mol Teratol. 2011;91:728–36. 21308978 10.1002/bdra.20770 PMC3429933

[44] Zhang FF, Cardarelli R, Carroll J, et al. Significant differences in global genomic DNA methylation by gender and race/ethnicity in peripheral blood. Epigenetics. 2011;6:623–9. 21739720 10.4161/epi.6.5.15335 PMC3230547

[45] Felling RJ. Song H Epigenetic mechanisms of neuroplasticity and the implications for stroke recovery. Exp Neurol. 2015;268:37–45. 25263580 10.1016/j.expneurol.2014.09.017 PMC4375064

[46] Li X, Xiao B, Chen XS. DNA methylation: a new player in multiple sclerosis. Mol Neurobiol. 2016;54:4049–59. 27314687 10.1007/s12035-016-9966-3

[47] Henderson-Smith A, Fisch KM, Hua J, et al. DNA methylation changes associated with Parkinson’s disease progression: outcomes from the first longitudinal genome-wide methylation analysis in blood. Epigenetics. 2019;14:365–82. 30871403 10.1080/15592294.2019.1588682 PMC6557551

[48] Jones MJ, Goodman SJ, Kobor MS. DNA methylation and healthy human aging. Aging Cell. 2015;14:924–32. 25913071 10.1111/acel.12349 PMC4693469

[49] Huo Z, Zhu Y, Yu L, et al. DNA methylation variability in Alzheimer’s disease. Neurobiol Aging. 2019;76:35. 30660039 10.1016/j.neurobiolaging.2018.12.003 PMC6436841

[50] Reho JJ, Guo DF, Morgan DA, Rahmouni K. mTORC1 (mechanistic target of rapamycin complex 1) signaling in endothelial and smooth muscle cells is required for vascular function. Hypertension. 2021;77:594–604. 33356400 10.1161/HYPERTENSIONAHA.120.14708 PMC8678954

[51] Thomis DC, Gurniak CB, Tivol E, et al. Defects in B lymphocyte maturation and T lymphocyte activation in mice lacking Jak3. Science. 1995;270:794–7. 7481767 10.1126/science.270.5237.794

[52] Wang YC, Cai D, Cui XB, et al. Janus kinase 3 deficiency promotes vascular reendothelialization-brief report. Arterioscler Thromb Vasc Biol. 2021;41:2019–26. 33910370 10.1161/ATVBAHA.121.316293 PMC8159884

[53] Stopa N, Krebs JE, Shechter D. The PRMT5 arginine methyltransferase: many roles in development, cancer and beyond. Cell Mol Life Sci. 2015;72:2041–59. 25662273 10.1007/s00018-015-1847-9 PMC4430368

[54] Motolani A, Martin M, Sun M, Lu T. The structure and functions of PRMT5 in human diseases. Life. 2021;11:1074. 34685445 10.3390/life11101074 PMC8539453

[55] Hamard PJ, Santiago GE, Liu F, et al. PRMT5 regulates DNA repair by controlling the alternative splicing of histone-modifying enzymes. Cell Rep. 2018;24:2643–57. 30184499 10.1016/j.celrep.2018.08.002 PMC6322662

[56] Piazza R, Magistroni V, Redaelli S, et al. SETBP1 induces transcription of a network of development genes by acting as an epigenetic hub. Nat Commun. 2018;9:2192. 29875417 10.1038/s41467-018-04462-8 PMC5989213

[57] Apostolidis SA, Stifano G, Tabib T, et al. Single cell RNA sequencing identifies HSPG2 and APLNR as markers of endothelial cell injury in systemic sclerosis skin. Front Immunol. 2018;9:359069. 10.3389/fimmu.2018.02191 PMC6174292 30327649

[58] Gotha L, Lim SY, Osherov AB, et al. Heparan sulfate side chains have a critical role in the inhibitory effects of perlecan on vascular smooth muscle cell response to arterial injury. Am J Physiol Hear Circ Physiol. 2014;307:H337–45. 10.1152/ajpheart.00654.2013 24858854

[59] Bang OY, Fujimura M. Kim S-K The pathophysiology of moyamoya disease: an update. J Stroke. 2016;18:12–20. 26846756 10.5853/jos.2015.01760 PMC4747070

[60] Sigdel TK, Shoemaker LD, Chen R, et al. Immune response profiling identifies autoantibodies specific to moyamoya patients. Orphanet J Rare Dis. 2013;8:45. 23518061 10.1186/1750-1172-8-45 PMC3648437

[61] Asselman C, Hemelsoet D, Eggermont D, et al. Moyamoya disease emerging as an immune-related angiopathy. Trends Mol Med. 2022;28:939–50. 36115805 10.1016/j.molmed.2022.08.009

[62] Review C, Communication S. Principles G World Medical Association Declaration of Helsinki: ethical principles for medical research involving human subjects. J Am Coll Dent. 2014;81:14–8. 25951678

