# Supplementary material for: Genome-Wide DNA Methylation Profiling Reveals Low Methylation Variability in Moyamoya Disease
Source: Transl Stroke Res. 2024 Oct 2;16(4):1198–213. doi: 10.1007/s12975-024-01299-w (PMC12202675; doi:10.1007/s12975-024-01299-w)
Supplement: Supplementary file 1 — Supplementary file1 (DOCX 1416 KB) [file 12975_2024_1299_MOESM1_ESM.docx]

**SUPPLEMENTARY MATERIALS**

**Genome-wide DNA Methylation Profiling Reveals Low Methylation Variability in Moyamoya Disease**

Kikutaro Tokairin, MD, PhD^1,2, †^; Masaki Ito, MD, PhD^1,2, †^; Alex G Lee, PhD^4, †^; Mario Teo, MD^1,2^; Shihao He, MD, PhD^3^; Michelle Y Cheng, PhD^1,2, *^; Gary K Steinberg, MD, PhD^1,2,*^

^1^Department of Neurosurgery, Stanford University School of Medicine, Stanford, CA, USA

^2^Stanford Stroke Center, Stanford University School of Medicine, Stanford, CA, USA

^3^Department of Neurosurgery, Peking Union Medical College Hospital, Peking, China

^4^Division of Hematology and Oncology, Department of Pediatrics, University of California, San Francisco, CA, USA

^†^Co-first authors.

^*^G.K.S and M.Y.C contributed equally to this work.

**Supplementary Table 1**

Binominal Test Across Multiple Cohorts


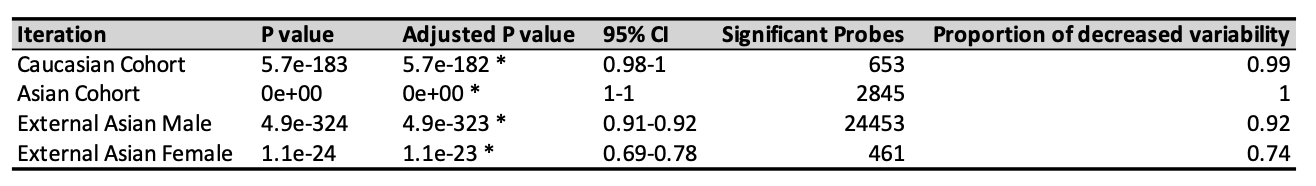


*All p-values were derived using two-tailed binomial tests. Adjusted p-values reflect Bonferroni correction for multiple testing across iterations.

**Supplementary Table 2**

Differential Variability Analysis Results from Iterative Subsampling of Caucasian Cohort


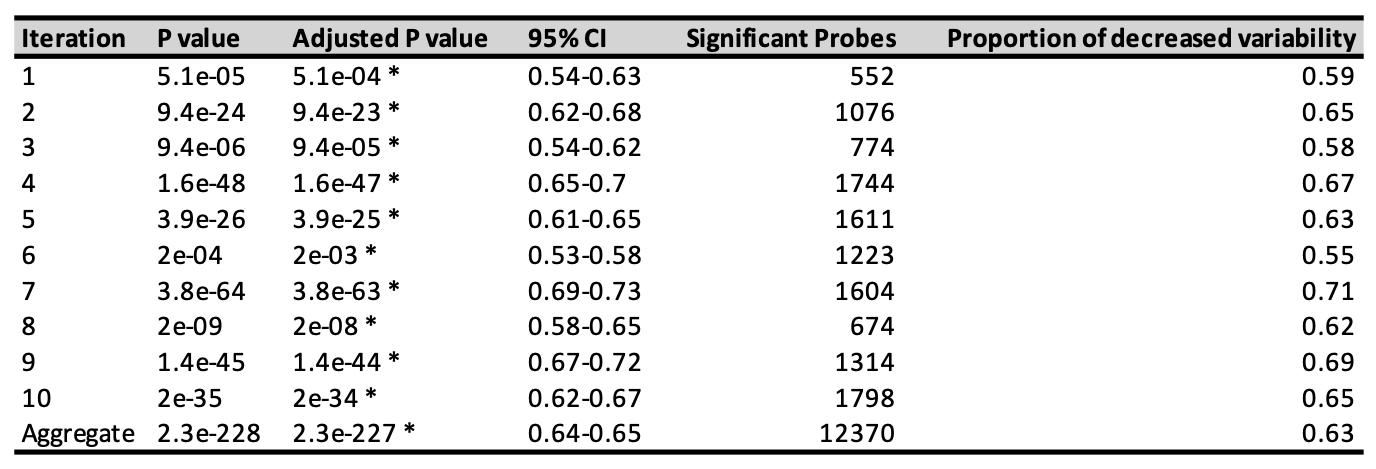


*All p-values were derived using two-tailed binomial tests. The aggregate row represents combined data from all iterations, with the proportion and confidence interval calculated from the pooled data. Adjusted p-values reflect Bonferroni correction for multiple testing across iterations.

**Supplementary Figure 1**


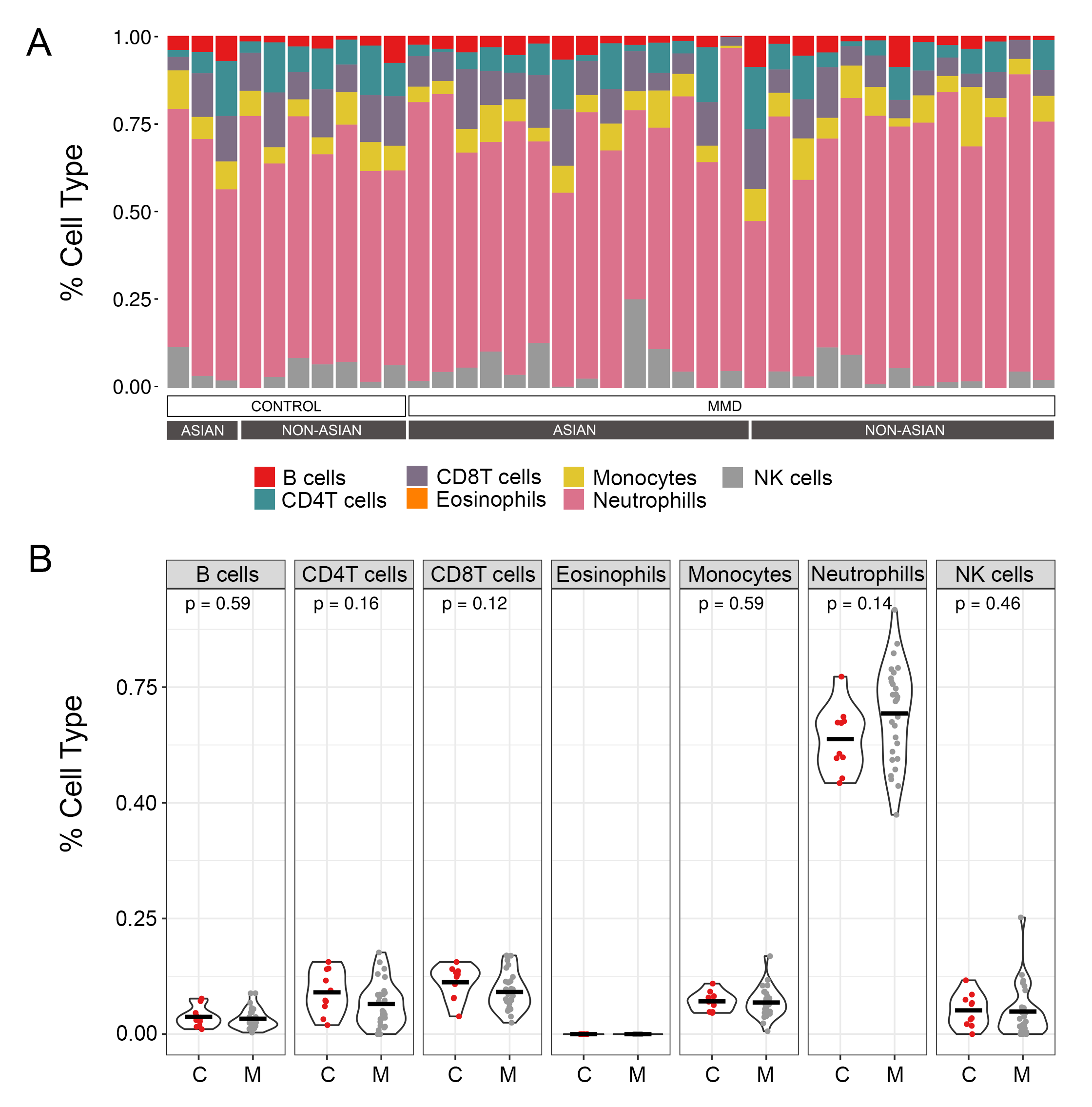


**Supplementary Figure 1.** **Cell type deconvolution analysis for all subjects in the Non-Asian and Asian cohorts.** **A**, Cell type proportions is shown for all study subjects in the control and MMD cohort: Non-Asian cohort (13 MMD patients and 7 healthy controls) and Asian cohort (14 MMD patients and 3 healthy controls). **B**, Cell type proportion was compared between control (C) and MMD (M) groups for each cell type by Wilcoxon signed-rank sum test followed by Bonferroni correction. Thick horizontal bar in the graphs represent the average of the cell type proportion for each group. There are no significant difference between MMD and control groups in the relative proportions of neutrophils, eosinophils, monocytes, B-cells, CD4+ T-cells, CD8+ T-cells or natural killer (NK) cells in the blood samples.

**Supplementary Figure 2**


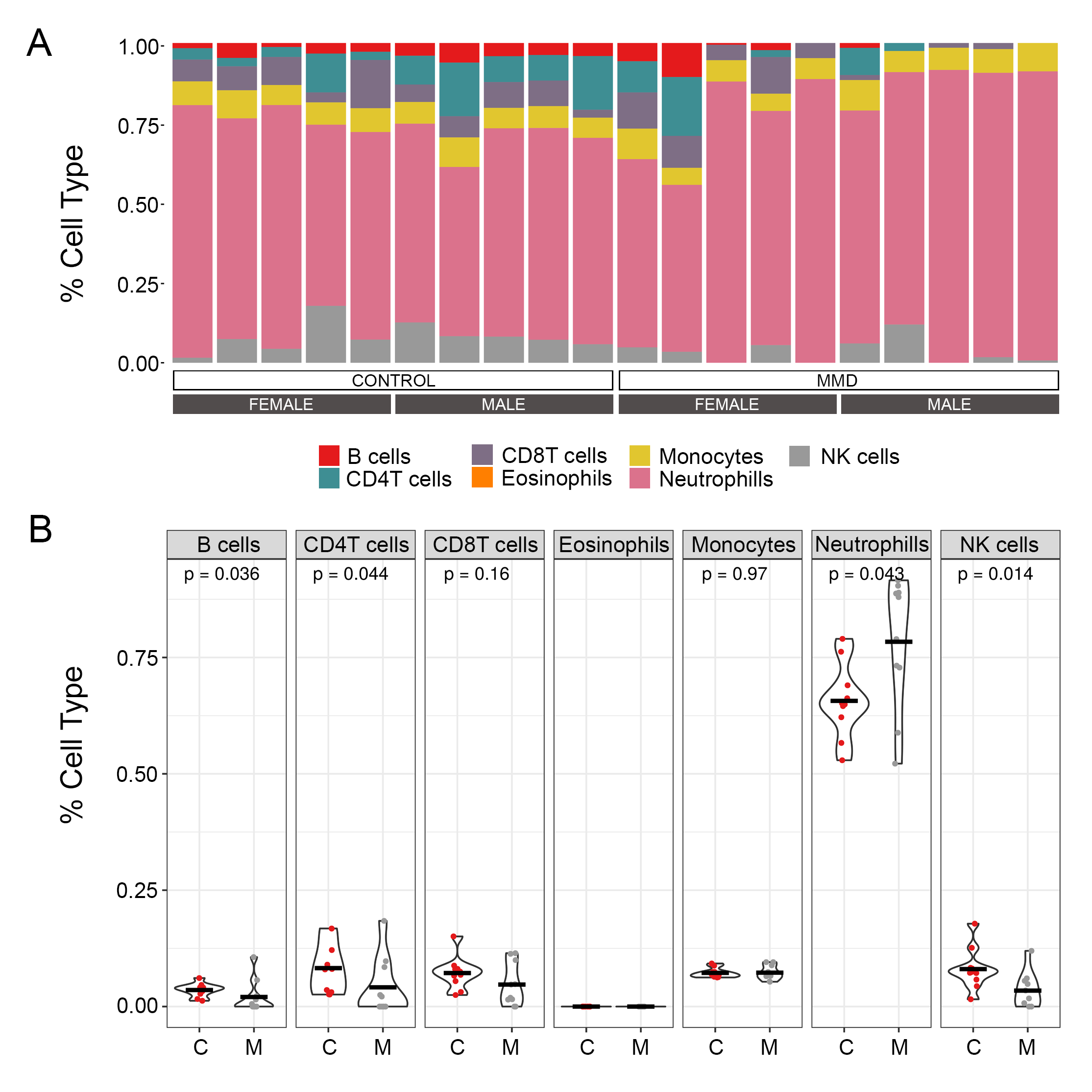


**Supplementary Figure 2.** **Cell type deconvolution analysis in all subjects of the external validation Asian cohort.** **A**, Cell type proportions is shown for all study subjects in the control (males n=5, females n=5) and MMD (males n=5, females n=5) cohort. **B**, Cell type proportion was compared between control (C) and MMD (M) groups for each cell type by Wilcoxon signed-rank sum test followed by Bonferroni correction. Thick horizontal bar in the graphs represent the average of the cell type proportion for each group. There are no significant difference between MMD and control groups in the relative proportions of neutrophils, eosinophils, monocytes, B-cells, CD4+ T-cells, CD8+ T-cells or natural killer (NK) cells in the blood samples.
